# Motor Performance in Male Youth Soccer Players: A Systematic Review of Longitudinal Studies

**DOI:** 10.3390/sports9040053

**Published:** 2021-04-19

**Authors:** Maryam Abarghoueinejad, Adam D. G. Baxter-Jones, Thayse Natacha Gomes, Daniel Barreira, José Maia

**Affiliations:** 1 Centre of Research, Education, Innovation, and Intervention in Sport (CIFI2D), Faculty of Sport, University of Porto, 4200-450 Porto, Portugal; dbarreira@fade.up.pt (D.B.); jmaia@fade.up.pt (J.M.); 2 College of Kinesiology, University of Saskatchewan, Saskatoon, SK S7N 5B2, Canada; baxter.jones@usask.ca; 3 Department of Physical Education, Federal University of Sergipe, São Cristóvão 49100-000, SE, Brazil; thayse_natacha@hotmail.com

**Keywords:** longitudinal, young, soccer players, motor performance

## Abstract

The aim of this systematic review was to identify and synthesize the available information regarding longitudinal data addressing young soccer players’ motor performance changes. Following the Preferred Reporting Items for Systematic and Meta-analyses (PRISMA) statement, literature searches were performed in three databases: PubMed, ISI Web of Science and SCOPUS. The following descriptors were used: football, soccer, youth, young, player, athlete, physical performance, motor performance, longitudinal. The inclusion criteria were original articles in English with longitudinal data of young males (aged 10–18 years), with the aim to investigate motor performance serial changes. The initial search returned 211 records, and the final sample comprised 32 papers. These papers covered the European continent, and used mixed and pure longitudinal design with variation in sample size and age range. The reviewed studies tended to use different tests to assess the motor performance and aimed to identify changes in motor performance in several ways. In general, they indicated motor performance improvements with age, with a marked influence of biological maturity, body composition, and training stimuli. This review highlights the need for coaches and stakeholders to consider players’ motor performance over time whilst considering biological maturation, biological characteristics, and training stimuli.

## 1. Introduction

Soccer is the world’s most popular sport and participants represent ~4.1% of the total sporting population [1]. With such large numbers of participants, governing bodies and other stakeholders invest significant amounts of money in soccer players’ talent identification. The identification and development of the next generation of young soccer players is a key goal for these organizations [2,3]. Thus, the design and implementation of appropriate programs to uncover youth soccer players’ potentials are common practice within soccer academies. These academies support the early development [4] and then the transition of young players into the senior professional world [5,6]. 

In a cross-sectional study, data are collected from many different individuals at a single time point and comparisons are made between different populations. In contrast, in a longitudinal study, the same data are collected in the same individuals over short or long periods of time. Therefore, whilst a cross-sectional study considers a snapshot in time, the longitudinal study design considers what happens before or after the snapshot is taken. The benefits of the cross-sectional design are that it allows researchers to compare many different variables at the same time. However, the disadvantage is that cross-sectional studies are not able to provide definitive information about cause-and-effect relationships. A longitudinal study can detect development or changes in population characteristics at both the group and individual level. Thus, longitudinal studies can establish sequences of events and enable the researcher to address cause-and-effect relationships. In youth soccer studies, the longitudinal design allows the researcher to distinguish the effects of training and competition from those associated with normal growth and development. There is much research devoted to describing and interpreting the manifold expressions of soccer players’ characteristics and their response to training and competition. Unfortunately, most available evidence is based on cross-sectional data [2], with few longitudinal reports or well-controlled experimental studies. This limits the current knowledge concerning youth soccer players’ development [7,8].

Recent systematic reviews of young soccer players have dealt with match running performance [9], talent identification [10], and anthropometric-physiological profiling [11]. These reviews identified a series of inconsistencies and gaps in the literature which have hampered practitioners’ abilities to make evidence-informed decision making [2,12,13]. Furthermore, there is an absence of research in young soccer players’ development processes such as the interactions of their physical growth and biological maturation with systematic training stimuli, estimation of velocities and spurts in their motor performance and specific skills’ levels, as well as players’ systematic responses to training and competition [8]. 

To the best of our knowledge, there apparently is no available systematic review dealing with young male soccer players’ longitudinal development of motor performance. Therefore, our goal is to provide a summary of existing longitudinal data dealing with male soccer players’ motor performance changes during adolescence, which is a very important time-window for the nurturing of soccer players’ careers. 

## 2. Materials and Methods

### 2.1. Protocol

This review used the “Preferable Reporting Items for Systematic Reviews and Meta-Analyses Protocols” (PRISMA-P) [14,15] to probe the literature of longitudinal studies into young soccer players’ motor performance. We also complied with the Cochrane Handbook for Systematic Reviews of Interventions (version 5.1.0; http://handbook-5-1.cochrane.org/, accessed on 10 October 2020). 

### 2.2. Information Sources and Search Strategy

The search strategy comprised two stages. First, the electronic databases MEDLINE (PubMed/PubMed Central interface), Web of Science™ Core Collection and SCOPUS were searched up to January 2021. The online search was performed based on the following strategy: (football OR soccer) AND (youth OR young OR player OR athlete) AND (physical performance OR motor performance) AND (longitudinal). Second, the reference list of the selected papers was searched for possible studies to be included in the review. A full description of the input arguments used in each database is also provided (Electronic Supplementary Material Table S1). EndNote software (version X9.0, X7.0.1, Clarivate Analytics, Philadelphia, PA, USA) was used as the citation manager during the processes of searching, deduplication, selection, and management of the studies.

### 2.3. Eligibility Criteria 

To be included in the review, original studies had to: (i) have a longitudinal design following players over time, i.e., with at least two repeated observations; (ii) have a sample of young male soccer players, i.e., athletes aged between 10 and 18 years; (iii) aim to investigate physical fitness/physical/motor performance and/or functional capacity (expressed by muscular strength and/or power, aerobic/anaerobic power, agility, flexibility, movement coordination and speed, as well as specific soccer technical performance such as dribbling and shooting, for example) serial changes; and (iv) be published in English and in peer-reviewed journals. Studies were excluded if: (i) psychological facets were mainly assessed, (ii) they used impaired players, and (iii) they concentrated on match-analysis. 

### 2.4. Study Selection

Two researchers (MA, TNG) independently conducted the online search. Grounded in the eligibility criteria, papers were firstly selected based on their title and abstract, and those selected had their full text screened. To be included in the present review, eligible papers had to be selected by the two researchers, and if any discrepancies were observed at this stage, reviewers discussed and resolved inclusion and/or asked for the judgement of a senior researcher (JM). The senior researcher examined each situation on a case-by-case basis and determined the inclusion or exclusion of a given article using his experience in the field. After the selection of the manuscript to be included, one of the researchers screened the reference lists of the selected papers to identify any other potential paper to be included in the review. Those studies selected in this stage were re-checked by the second researcher, and only those approved by both were considered for inclusion in the present study. 

### 2.5. Methodological Quality Assessment

The quality of the included articles was assessed with the modified version of current established scale used in sport science, health care and rehabilitation (i.e., Cochrane, Coleman, Delphi, and Physiotherapy Evidence Database (PEDro)). The current scale (Table 1) was adapted from a recent review by Sarmento et al. [16]. Articles were assessed based on their purpose (Q1), participants’ characteristics (Q2), sample justification (Q3), motor performance assessments (Q4), statistical procedures used (Q5), results and outcome (Q6), study method conclusion (Q7), practical implications (Q8), limitations (Q9), and future direction (Q10). All ten quality criteria were scored on three levels (2-point per item), i.e., a score of zero (no), one (maybe), and two (yes) given for each item. The total scores ranged between zero and twenty. A sum of scores from all questions was subsequently computed. To make a fair comparison between studies with different designs, the decision was made to calculate a percentage score as a final measure of methodological quality. For this, the total score was converted into percentages, ranging from 0 to 100%, to ensure that the quality assessment was equitable across all the included articles. Studies were categorized into 3 levels; high (≥75%), moderate (50–74%) and low (<50%) methodological quality scores [16]. Methodological quality was not evaluated for the purpose of including/excluding studies. Two researchers (MA, TNG) performed independent assessments. If discrepancies occurred, these were resolved in a consensus discussion with third senior researcher (JM). 

### 2.6. Strategy for Data Synthesis

A descriptive synthesis of the findings from the included studies is presented in Table 2, where summaries with reference to authors and years of publication were provided. Then, the terminologies used in motor performance variable definition and assessment were examined. Demographic details were extracted, including sample size, age/age group of participations, and the geographical location of the players. Design aspects (mixed-longitudinal, longitudinal), configuration (duration), and measurement techniques/equipment were also included. Finally, general results regarding changes in motor performance were extracted and main findings were organized and described.

## 3. Results

### 3.1. Included Studies

**Figure 1:**
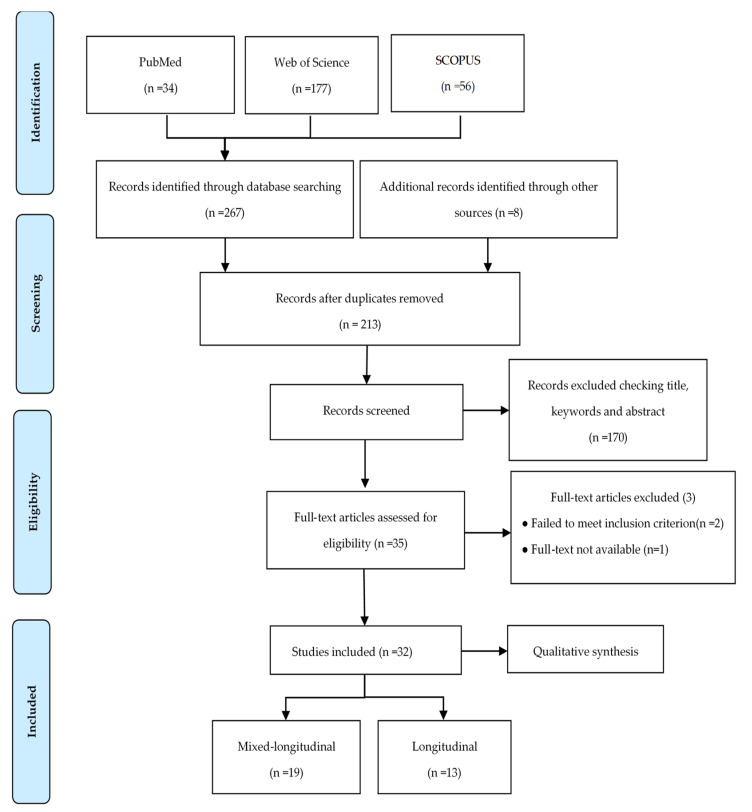
Flow chart including literature search and selection steps following the Preferred Reporting Items for Systematic and Meta-analyses (PRISMA) statement.

Study collection database searches retrieved 267 citations. Figure 1 shows the number of articles found in each electronic database and the literature search/selection processes, including all the steps performed. After exclusion of duplicates, two hundred and five articles remained, and eight additional articles identified through other sources were included in the selection process. The remaining 213 articles were screened based on their title and abstract, and one hundred and seventeen articles were excluded at this stage. The remaining 35 studies were screened for full text assessment. One study did not have its full text available, and two other articles were excluded since they did not precisely examine the development of motor performance variables. Thirty-two articles fulfilled all the inclusion criteria and were chosen at the end of the screening procedure for the in-depth analysis (i.e., qualitative analysis) and review. 

### 3.2. Methodological Quality

Quality scores attributed to studies are found in Table 2 and in the Electronic Supplementary Materials (Table S2). The quality of indicators was as follows: (i) the mean ± standard deviation score of the 32 articles was 79 ± 10 percent; (ii) none of the studies achieved the maximum score of 100% or scored below 50% (low quality); (iii) eight articles were classified as of moderate quality (ranging between 51 and 75%) [17,18,19,20,21,22,23,24], but (iv) twenty-four received high methodological quality scores (>75%). Putative deficiencies were mostly related to question 3 (justification of the study sample size), and question 9 (limitations of the study acknowledged).

### 3.3. Studies’ Characteristics

#### 3.3.1. Location

**Figure 2:**
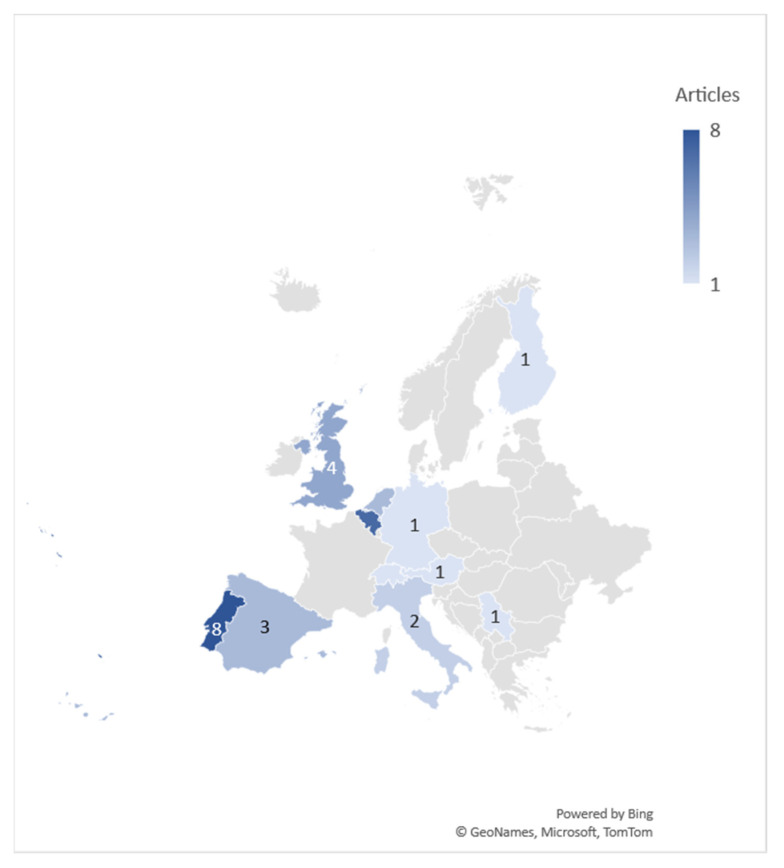
Number of studies by country.

All studies were from the European continent: eight were conducted in Portugal (25.8%) [19,22,25,26,27,28,29,30], seven in Belgium (22.6%) [17,23,31,32,33,34,35], four in the United Kingdom (12.9%) [36,37,38,39], three in the Netherlands (9.7%) [18,40,41], three in Spain (9.7%) [24,42,43], two in Italy (6.5%) [20,44]. Single studies were conducted in Austria [45], Finland [46], Germany [47], Switzerland [21], and Serbia [48] (Figure 2). 

#### 3.3.2. Sample Size and Design 

**Figure 3:**
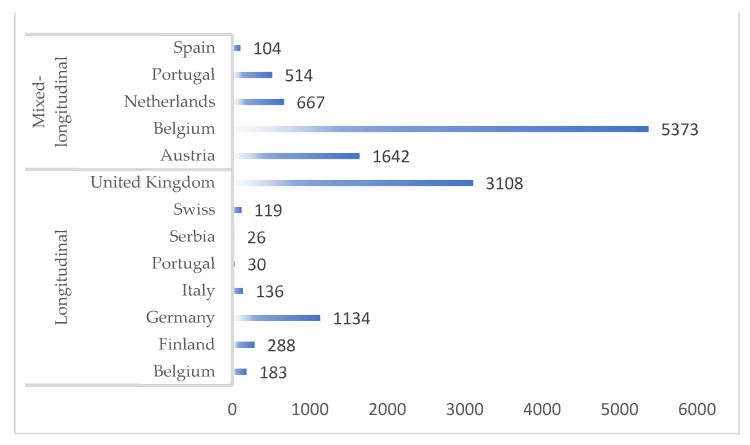
Total number of subjects across studies by country.

Motor performance was investigated in a total of 12,190 youth soccer players, representing an average of ~380 players per study. Nineteen studies used a mixed-longitudinal design, with sample sizes ranging between 16 [22] and 2228 [35], and age ranging from 5 to 20 years. Time durations (serial data collection) ranged from three [41] to nine years [45]. Thirteen studies used a pure longitudinal design lasting from ten weeks [30] to eleven years of a prognostic period [38]. Sample sizes varied from 6 [37] to 2875 subjects [38], and player age ranged from 7 [20] to 19 years [38] (Figure 3).

#### 3.3.3. Motor Performance Assessments (Tests)

Motor performance, soccer-specific motor performance, and soccer-specific skills were distinctively assessed. Twenty-five studies [17,18,19,20,21,22,23,24,25,26,27,29,30,31,33,34,35,36,37,38,39,43,44,45,46,47] used tests such as plate tapping, sit and reach, sit-ups, bent arm hang, standing long jump, vertical jump with and without free arm, endurance shuttle run, sprints (10, 15, 20, and 30 m), medicine ball throw 2 kg, multi-stage endurance run, agility (505 test, barrow zigzag run, 8-figure, T-Test hurdles run, slalom course, slalom running with obstacles) and the multistage fitness tests. Seventeen studies [17,18,19,21,26,27,28,29,31,32,35,36,40,42,43,45,48] assessed soccer-specific motor performance, namely: 30 m repeated sprint (RSA), agility shuttle run 5 × 10 m (SHR), intermittent endurance (ISRT), the Yo-Yo Intermittent Recovery Tests, slalom sprint and shuttle sprint. Additionally, a dozen [18,20,21,22,27,28,29,38,41,44,46,47] of them also assessed soccer-specific skills including dribbling, dribbling with a pass, shooting, shooting accuracy, ball control, touch of the ball with the body and the head, juggling, passing, and wall pass.

#### 3.3.4. Changes in Motor Performance

Overall, the reviewed studies aimed at identifying changes in motor performance in different ways: (i) modelling mean trends as well as their covariates [18,19,22,23,25,26,27,28,29,31,33,34,38,40,41,42,43,47]; (ii) describing mean changes over time [20,30,32,35,36,39,44,45,46,48]; (iii) aligning changes by age-at-peak height velocity [37,43]; (iv) identifying timings of spurts in different motor performance tests [17]; and (v) describing patterns of change [21].

Most multilevel/mixed modelling with polynomial age trends (age, age^2^, and age^3^) showed systematic increases in soccer players’ aerobic capacity [25,31,40,42]; however, two did not [22,38]. Training stimuli [25,40] and playing position [38] were linked to aerobic capacity differences, except for goal-keepers [22]; maturity status was not related to these trends [31,42]. There is evidence [24,33,34,38] for lower limb explosive strength (counter-movement jump) increasing non-linearly with increasing age, while the increase is linear in standing broad jump test [23]. These increases were related to maturity status [24,34], fat-free mass [34], playing position [38], and previous performance [23]. There are also reports [24,28,29,38,43,47] showing non-linear improvements in change in direction, which were explained by training stimuli [24], fat-free mass [28,29], and playing position [28,38]. 

Most straight speed [24,38,43,47] and repeated sprint ability [19,26] showed non-linear trends, although one showed a linear trend [24]. Maturity status [24], fat-free mass, and playing position [38] were associated with these changes. Additionally, training stimuli, lower limb explosive strength, and fat-free mass were identified as additional repeated sprint covariates [19,26]. Furthermore, there was also evidence that future professional players had systematic higher physical performance levels than future non-professionals [38,47]. Additionally, non-linear trends were observed in soccer technical skills [22,28,29,41,47]. Players with more training stimuli [22,28,29] and more lower limb explosive strength [29] and midfielders [28] were better regarding dribbling speed.

During soccer seasons, significant differences were evident in motor performance [20,35,39,45]. However, one study did not identify such changes in different age groups [39]. There is also evidence that motor performance remained relatively high and stable during the period of one year [46], and in particular, aerobic capacity showed high stability over two years and moderate stability over four years [32]. Three years of training was associated with changes in physical performance independent of baseline levels and maturational change [36]. Ten weeks of physical and tactical training in small-sided games produced a moderate effect on U15 change in direction, moderate improvements in U17 lower limb explosive strength, and a positive effect on attackers’ physical performance [30]. In contrast, one study reported that a season follow-up improved U14 players’ motor performance independent of training stimuli [44]. 

Two studies aligned motor performance changes with age-at-peak height velocity (PHV) [37,43]. A case study reported systematic fluctuations in players’ straight speed and lower limb explosive strength performance [37]. On the other hand, the maximum velocity of lower limb explosive strength occurred 2 years after PHV, straight speed was coincident with PHV, whereas change in direction and aerobic capacity started levelling off their increases 3–4 years after PHV [43]. Contrarily, one study showed that almost all physical performance peak spurts occurred at PHV and that a plateau in straight speed, lower limb explosive strength and upper-body endurance development occurred after PHV [17]. Finally, one study used a person-centered approach aiming to identify players’ patterns of change and showed partial structural stable clustering as well as high individual stability [21].

## 4. Discussion

In this systematic review, our aim was to provide a comprehensive overview of the current body of evidence of existing longitudinal research concerning young soccer players’ motor performance. Across studies, there is evidence of motor performance improvements with chronological age, as well as marked influences of biological maturity, body composition and training stimuli. Further, researchers based their analyses and conclusions on data from pure longitudinal and mixed-longitudinal designs. Notably, all studies sampled European adolescent players. 

### 4.1. Study Quality

Overall, studies tended to adhere to high quality standards. Yet, a less favorable point is related to the apparent absence of sample size justification and a putative insufficiency of this aspect is evident when discussing results’ generalization. This, in turn, may weaken to a certain degree their external validity [49]. In any case, it is also important to consider pragmatic factors and/or research design requirements when sampling players and have their regular assessments which are often conditioned by their training schedules and academic obligations. This is a viable argument when there is a need for systematic and highly regular assessments [50]. As such, we suggest that future studies should discuss potential flaws of their designs, especially sample size, as well as ways of adequately dealing with missing data [51] before drawing conclusions about the results’ transferability to other settings, namely coaches’ decisions when planning their training sessions as well as their expectations. 

### 4.2. Location 

Although one important aim of the grassroots FIFA program focuses on “Develop the game” for all [52], there apparently is no doubt that appropriate nurturing of young soccer players is time- and money-consuming, as well as being a challenging process [53]. The studies retrieved in this systematic review are from European countries that received some form of funding from their governmental agencies. Furthermore, not only did their progressive governments’ sport policies incorporate elements of soccer grassroots programs [54,55], but this is also in the interest of coaches and managers from private soccer clubs. We suggest that future longitudinal research with young soccer players should also be conducted worldwide. This requires, of course, the presence of collaborative research teams from different countries and continents, linking soccer producer countries with those apparently less advanced in terms of research, team building, and soccer education. 

### 4.3. Motor Performance Assessments 

Physical performance tests offer objective assessments of young soccer players that can generally be used for different purposes—description of systematic changes and their covariates, selection and placement, assess individual progress, i.e., diagnostics, prediction, and evaluation of training intervention programs [56]. Most reviewed papers dealt with the description of mean changes in important physiological markers such as aerobic capacity, lower limb explosive strength, and speed, by the use of different tests. A similar trend was observed for soccer-specific physical performance and technical skills. In general, technical skills improved with chronological age, as expected from players’ regular training schedules. Even though tests were different for measuring the same construct across studies, similar results were identified and may be linked (i) to the fact that tests were appropriate for the age range and sample characteristics, and (ii) to expected changes during adolescence as part of their natural developmental course plus the systematic and cumulative effects of training and competition. 

It was found that soccer’s physiological demands and technical skills are different for goalkeepers and outfield players [57], and this is probably the main reason why most studies [18,25,28,41] decided to not include goalkeepers during data sampling and/or their analysis. However, Rebelo-Gonçalves et al. [22] sampled 16 goalkeepers that were similarly tested (aerobic capacity, dribbling and passing skills) as their outfield players peers. However, in a previous study [28], using the sample from the same research project from Rebelo-Gonçalves et al. [22], the authors decided to exclude goalkeepers during sample selection/data analysis because the sample size was very small. Hence, we emphasize the need for future research to direct its goal to goalkeepers’ physical performance characteristics and as well as their specific technical skills.

### 4.4. Statistical Procedures and Changes in Motor Performance 

There apparently is no specific trend in statistical procedures used to analyse motor performance changes across studies’ publication years. Most studies [18,19,22,23,25,26,27,28,29,31,33,34,39,40,41,42,43,47] used multilevel/mixed modelling independent of study design, duration, and sample size. In general, they relied on polynomials of age (age, age^2^, age^3^) to estimate motor performance mean trends (linear and non-linear), as well as adding different predictors of such trends, namely training stimuli and maturity status [22,24,25,28,29,34,40], and reported their different effect sizes. 

When focusing on mean changes across time, there apparently is no parallel trend across studies. For example, when using independent factors as group—control versus experimental [48]—or players’ levels [30,45], training effects on motor performance as well as its stability vary by using different statistical methods such as analysis of variance [30,37] or the general linear model [35]. Yet, we were not able to localize a study that investigated the tracking of players’ motor performance, notwithstanding the fact that stability of changes was mentioned [32,46]. One study [17] used a non-smoothed polynomial method to identify spurts in several physical performance markers aligned by age-at-PHV, and showed that in spite of their different intensities, they tend to peak around PHV. Another study used a person-centered approach with a cluster analysis to describe players’ patterns of change and obtained partial structural stable clustering along with high individual stability [21]. One case study [37] showed that physical performance trajectories are irregular, occurring quickly and in a radical fashion, suggesting that individual differences between soccer players tend to be temporary. We concur with the authors that there is a novel need to longitudinally investigate young players as single cases, aiming to gain a better understanding of their erratic and systematic changes in order to assist coaches when structuring their training program as well as when making selection decisions. 

There is a strong suggestion that motor performance changes are related to biological maturation differences, between and within players, as well as their training stimuli. Yet, there apparently is no unequivocal proof of the effects of different training interventions and bio-banding [58] on players’ motor performance. Therefore, we recommend additional research for a deeper understanding of the impact of training interventions on motor performance during puberty, especially their hormonal and physiological mechanisms. Additionally, we could not find a theoretical basis for conducting research apart from using ANOVAs or the multilevel/mixed model. We contend that future research should also consider players’ contexts, i.e., their families, coaches, and clubs. Young players’ development occurs within these contexts and they should be acknowledged. In sum, there is a need to also use multidimensional and/or ecological approaches to enhance our understanding of the complexities of young players’ development [8]. 

### 4.5. Limitations of the Current Review

This is most probably the first systematic review on young male soccer players’ motor performance development based on serial data (pure longitudinal and mixed-longitudinal), and it is not without limitations. First, it is possible that the retrieved publications are not free from bias towards positive results. As such, we suggest future studies to combine available data for meta-analyses with proper statistical evaluation of publication bias. Second, we restricted our criteria to only include male players. We urge future research to also consider female players’ motor performance serial data. Third, it is also possible that the review criteria and search strategy may have limited our scan. Fourth, although no study used in this systematic review reported injuries or orthopedic problems, care must be taken when interpreting data because of a putative equinus condition [59].

In spite of these limitations, we tried to present a comprehensive description of available longitudinal data during players’ puberty given that it is considered a very important time window that may likely benefit soccer stakeholders to employ better developmental sporting strategies in their organizations to maximize young soccer players’ potentials and smooth their career transitions.

## 5. Conclusions

The present study compiled current empirical evidence on longitudinal data dealing with male soccer players’ motor performance changes during puberty. Puberty has been found to be a crucial time for nourishing soccer players’ future quality vocations. Amongst studies, it was observed that motor performance improved with chronological age, which was linked to biological maturity, body composition changes and training stimuli. Coaches and stakeholder of young soccer players should be aware of the positive influence of physical and biological maturation, training stimuli and systematic fluctuations on players’ immediate motor performance. This suggests that selection and deselection decisions should be made based on longitudinal rather than cross-sectional information. We propose that future longitudinal studies with young soccer players should also be global, with a focus on playing position, cases study, tracking methods, and deeper understanding of the impact of training interventions on motor performance during puberty, especially their hormonal and physiological mechanisms. Finally, there is a need for more research on the contextual and environmental aspects impacting motor performance development. 

## Figures and Tables

**Table 1:** Methodological quality scoring system (adapted from Sarmento et al., 2018 [16]).

	Question	Answer	Score
Q1	Was(were) the aim(s) of study clearly set out?	Yes = 2; Maybe = 1; No = 0	0–2
Q2	Were characteristics of participants presented in detail in methods section? (number of subjects, sex, age, country/city)	Yes = 2; Maybe = 1; No = 0	0–2
Q3	Was sample size justified?	Yes = 2; Maybe = 1; No = 0	0–2
Q4	Are the motor performance to be measured clearly described in the methods section?	Yes = 2; Maybe = 1; No = 0	0–2
Q5	Were statistics clearly presented?	Yes = 2; Maybe = 1; No = 0	0–2
Q6	Resultsʹ details (means and standard deviations and/or change/ difference, effect size/mechanistic magnitude-based inference)	Yes = 2; Maybe = 1; No = 0	0–2
Q7	Were conclusions appropriate given the study methods and the objectives?	Yes = 2; Maybe = 1; No = 0	0–2
Q8	Are there any implications for practice given the results of the study?	Yes = 2; Maybe = 1; No = 0	0–2
Q9	Were limitations of the study acknowledged and described by the authors?	Yes = 2; Maybe = 1; No = 0	0–2
Q10	Are there any future direction described by the authors?	Yes = 2; Maybe = 1; No = 0	0–2
Total			0–20

Strict rules applied (No information = 0 point; 1–2 items described = 1 point; all items described = 2 points).

**Table 2:** Characteristics of studies included in the review.

Author/Country	Study Design Duration	Participants		Motor Performance Assessments (Tests)	Main Results	Quality Score
		Age Number				
(Philippaerts et al., 2006) [17]/Belgium	Mixed-longitudinal 5 y	11–13 y at baseline	33	Physical performance: Plate tapping, sit and reach (SR), sit-ups, bent arm hang, standing long jump (SLJ), vertical jump (VJ), endurance shuttle run (ESHR). Soccer specific physical performance: 30 m repeated sprint (RSA), agility shuttle run 5 × 10 m (SHR).	Physical performance improved non-linearly and reached its peak around peak height velocity (PHV), yet with different timing and tempo.	65%
(Huijgen et al., 2010) [18]/Netherlands	Mixed-longitudinal 5 y	12–19 y at baseline	267	Physical performance: shuttle sprint and dribble test. Soccer specific skills: slalom sprint and dribble test.	Speed and dribbling improved with age mainly at 12–14 y, but with different tempo. Dribbling improved after 16 y and sprinting from 14 to 16 y. Additionally, fat free mass, weekly hours of practice and playing position were positively associated with dribbling changes.	70%
(Mirkov et al., 2010) [48]/Serbia	Longitudinal 4 y	11 y at baseline	S_g = 26 C_g = 63	Physical performance: SR, SLJ, countermovement jumps (CMJ) with and without arm swing, slalom running with obstacles, SHR.	Physical performance improved with age in both groups, yet soccer players performed better in agility and motor coordination.	85%
(Roescher et al., 2010) [40]/Netherlands	Mixed-longitudinal 5 y	12–19 y at baseline	Pro = 53 N_pro = 77	Soccer specific physical performance: intermittent endurance (ISRT).	Aerobic capacity increased non-linearly with age but differences between groups occurred from 17 y onwards. Training was positively associated with performance.	75%
(Williams et al., 2011) [39]/United Kingdom	Longitudinal 3 y	U12–U16 at baseline	200	Physical performance: sprints 10 m (S10 m), 30 m (S30 m), VJ.	Physical performance improved linearly but with different rates for 10 m speed, 30 m sprint and vertical jump.	80%
(Gonaus and Muller, 2012) [45]/Austria	Mixed-longitudinal 9 y	14–17 y at baseline	1642	Physical performance: S20 m, hurdles agility run, CMJ, drop jump, foot tapping reaction medicine ball throw 2 kg, SR, 20 m multi-stage endurance run (MSER). Soccer specific physical performance: (SHR).	Speed, power, flexibility, and endurance improved with age. Power and flexibility as well as endurance effect sizes decreased with age; however, in speed results were stable from 14 to 17 y.	80%
(Valente-dos-Santos et al., 2012) [25]/Portugal	Mixed-longitudinal 5 y	11–13 y at baseline	83	Physical performance: MSER.	Aerobic performance unfolding was related to chronological and skeletal ages, and training stimuli.	90%
(Valente-dos-Santos et al., 2012) [19]/Portugal	Mixed-longitudinal 5 y	11–13 y at baseline	83	Physical performance: MSER, CMJ. Soccer specific physical performance: RSA.	Repeated sprint performance changes were related to chronological and skeletal ages, as well as fat free mass, aerobic endurance, and lower limb explosive strength.	55%
(Valente-dos-Santos et al., 2012) [26]/Portugal	Mixed-longitudinal 5 y	11–13 y at baseline	83	Physical performance: MSER, CMJ. Soccer specific physical performance: RSA.	Repeated sprint performance development was related to chronological age, maturity status, fat free mass, body size, aerobic endurance, and lower limb explosive strength and annual training.	80%
(Valente-dos-Santos et al., 2012) [27]/Portugal	Mixed- Longitudinal 5 y	11–13 y at baseline	83	Physical performance: SHR, MSER, CMJ. Soccer specific physical performance: RSA. Soccer specific skills: Ball control, dribbling speed, Shooting accuracy, wall pass.	Overall physical performance development was related to chronological age, maturation status, fat mass, dribbling speed and training stimuli. In general, soccer skills unfolding was related to chronological age, playing position, fat and fat-free mass, repeated sprint and aerobic endurance and training stimuli.	90%
(Huijgen et al., 2013) [41]/Netherland	Mixed-longitudinal 3 y	10–18 y at baseline	270	Soccer specific skills: Loughborough Soccer Passing (LSPT).	Soccer skills improved non-linearly: 18% in speed pass, and 32% in speed and accuracy pass with age.	85%
(Carvalho et al., 2014) [42]/Spain	Mixed- Longitudinal 4 y	U11 age category at baseline	33	Soccer specific physical performance: The Yo-Yo Intermittent Recovery Test (YYIR1).	Aerobic performance increased non-linearly with chronological age; yet, between 12–13 y decreased. Additionally, aerobic performance was related to training stimuli but not with body size and maturity status.	80%
(Deprez et al., 2014) [31]/Belgium	Longitudinal 5 y	11–14 y at baseline	162	Soccer specific physical performance: YYIR1.	Aerobic performance improved non-linearly with age and was related to stature, fat-free mass, and motor coordination.	85%
(Valente-dos-Santos et al., 2014) [28]/Portugal	Mixed-longitudinal 5 y	10–14 y at baseline	83	Physical performance: SHR. Soccer specific skills: Dribbling.	Agility development was related to chronological and skeletal age, stature, fat-free mass and playing position. Dribbling changes were related to chronological and skeletal age, stature, playing position and training stimuli.	85%
(Valente-dos-Santos et al., 2014) [29]/Portugal	Mixed-longitudinal 5 y	11–13 y at baseline	83	Physical performance: SHR, MSSE, CMJ. Soccer specific skills: Dribbling.	Agility changes were related to skeletal age, maturity status, fat-free mass, aerobic endurance, and explosive strength. Dribbling changes were associated with skeletal age, maturity states, fat-free mass, aerobic endurance, explosive strength, and training stimuli.	75%
(Wrigley et al., 2014) [36]/United Kingdom	Longitudinal 3 y	U12–U16 age category at baseline	S_g = 27 C_g = 18	Physical performance: S10 m, S20 m, CMJ, agility (505 test). Soccer specific physical performance: RSA, YY IRT2.	Systematic soccer specific training stimuli had significant effects on physical performance changes in young male players independently from baseline levels of performance and biological maturation.	90%
(Bidaurrazaga-Letona et al., 2015) [24]/Spain	Mixed- Longitudinal 4 y	U11 age category at baseline	38	Physical performance: CMJ, agility (barrow zigzag run), S15 m.	Non-linear improvement in explosive strength and agility performance with higher development rates for early matures. However, late matures had better linear improvements in speed performance.	75%
(Deprez et al., 2015) [32]/Belgian	Longitudinal 2 y and 4 y	4 y: ~12 y at baseline 2 y: ~13 y at baseline	21 21	Soccer specific physical performance: YYIR1.	Aerobic performance stability was moderate in 4 y and high over 2 y.	85%
(Deprez et al., 2015) [33]/Belgian	Mixed- Longitudinal 7 y	7–17 y at baseline	555	Physical performance: CMJ, standing broad jump (SBJ).	Explosive strength development was related to chronological age and motor coordination. However, in 11–15 y was positively influenced by stature and negatively by fat mass, but in 16–20 y positively influenced by fat free mass.	75%
(Deprez et al., 2015) [34]/Belgian	Mixed- Longitudinal 7 y	11–14 y at baseline	356	Physical performance: CMJ, SBJ.	Explosive strength performance improved non-linearly with age in CMJ test and linearly is SBJ. Additionally, explosive strength performance was related to leg length, fat free mass, flexibility, and motor coordination also maturity status except in SBJ test.	90%
(Forsman et al., 2016) [46]/Finland	Longitudinal 1 y	12–14 y at baseline	288	Physical performance: S30 m, agility (8-figure). Soccer specific skills: Dribbling, passing.	Physical performance and soccer skills remained relatively high and stable across the period of one year.	85%
(Francioni et al., 2016) [20]/Italy	Longitudinal one season	U8-U12 age category at baseline	103	Physical performance: CMJ with and without free arm, S15 m. Soccer specific skills: Touch of the ball with the body and the head, passing, shooting, dribbling, dribbling with pass.	Physical performance and soccer specific skills increased with age in one season.	70%
(Zuber et al., 2016) [21]/Swiss	Longitudinal 3 y	U13 age category at baseline	119	Physical performance: S40 m, CMJ. Soccer specific physical performance: YY IRT1. Soccer specific skills: Dribbling, passing, Juggling.	Change pattern showed to be partial structural with high individual motor performance stability.	70%
(Carvalho et al., 2017) [43]/Spain	Mixed- Longitudinal 6 y	U11 age category at baseline	33	Physical performance: Agility (barrow zigzag run), S15 m, CMJ. Soccer specific physical performance: YYIR1.	Agility and aerobic performance improved non-linearly and reach a steady rate around 3–4 y after PHV. Sprint and explosive strength maximum velocity occurred around 2 y after PHV.	80%
(Fransen et al., 2017) [35]/Belgian	Mixed- Longitudinal 6 y	5–20 y at baseline	2228	Physical performance: Agility (T-Test), S10 m, S20 m, S30 m, SR, hand grip. Soccer specific physical performance: YYIR1.	Linear increases of all physical performance tests. Yet, there is a suggestion of reaching a plateau around 15–17 years of age.	75%
(Rebelo-Goncalves et al., 2017) [22]/Portugal	Mixed- Longitudinal 5 y	11–13 y at baseline	16	Physical performance: MSSE. Soccer specific skills: Dribbling speed, wall pass.	Aerobic capacity and passing skills improved linearly in goalkeepers yet dribbling speed development was non-linear. Soccer skills improvement were mostly explained by training stimuli not by fat-free mass increases.	60%
(Francioni et al., 2018) [44]/Italy	Longitudinal one season	U14 age category at baseline	33	Physical performance: CMJ with and without free arm, S15 m. Soccer specific skills: Touch of the ball with the body and the head, passing, shooting, dribbling, dribbling with pass.	Motor performance improved in U14 age categories during one soccer season independent of training exposure.	80%
(Coutinho et al., 2018) [30]/Portugal	Longitudinal 10 weeks	U15, U17 age category at baseline	E_g = 15 C_g = 15	Physical performance: CMJ, S30 m, agility (repeated change in direction).	Physical performance of U15E improved in 10 weeks. Training had a moderate effect in U15E agility and in U17E CMJ improvements.	95%
(Leyhr et al., 2018) [47]/Germany	Longitudinal 3 y	U12 age category at baseline	1134	Physical performance: S20m, agility (slalom course). Soccer specific skills: Dribbling, ball control, shooting.	Motor performance improved non-linearly with time. Future professional players performed better at baseline and maintained their superiority across time.	80%
(Bennett et al., 2019) [23]/Belgium	Mixed-longitudinal	6–20 y at baseline	2201	Physical performance: CMJ, SBJ.	Explosive strength improved non-linearly with age. The length of the time between assessments did not show a strong impact on player’s future performance.	70%
(Moran et al., 2020) [37]/United Kingdom	Longitudinal 6 y	U10 age category at baseline	6	Physical performance: S10 m, S20 m, CMJ.	Straight speed and lower limb explosive strength performance can arise rapidly and in radical fashions.	90%
(Saward et al., 2020) [38]/United Kingdom	Longitudinal 11 y	U9–U19 age category at baseline	2875	Physical performance: S20 m, agility (slalom test), CMJ, the multistage fitness tests/ 20 m multi (MSER) (MSFT).	Agility, explosive strength, and speed improved non-linearly except aerobic capacity which improved linearly with age. Differences in playing position occurred in physical performance development. Future professional players had a faster rate as they get older, with different development patterns in explosive strength and agility.	90%

y = years, g = group, Pro = professional, S = soccer, C = control, E = experimental.

## Data Availability

Not applicable.

## Supplementary Materials

The following are available online at https://www.mdpi.com/article/10.3390/sports9040053/s1, Table S1: Full search strategy for each database with arguments presented as they were used, Table S2: Scores attributed to each study according to twelve criteria used in evaluating methodological quality.
