# Supplementary material for: Motor Performance in Male Youth Soccer Players: A Systematic Review of Longitudinal Studies
Source: Sports (Basel). 2021 Apr 19;9(4):53. doi: 10.3390/sports9040053 (PMC8072970; doi:10.3390/sports9040053)
Supplement: Supplementary file 1 [file sports-09-00053-s001.zip › Electronic Supplementary Material Table S1_MaryamA.pdf]

**Electronic Supplementary Material Table S1**. Full search strategy for each database with arguments presented as they were used.
**PubMed**
("football"[All Fields] OR "soccer"[All Fields]) AND ("youth"[All Fields] OR "young"[All Fields] OR "player"[All Fields] OR "athlete"[All Fields]) AND ("motor performance"[All Fields] OR "physical performance"[All Fields]) AND "longitudinal"[All Fields]
**Web of Science**
((football OR soccer) AND (youth OR young OR player OR athlete) AND (motor performance OR physical performance) AND (longitudinal)) - Refined by "Document Types": Article
**SCOPUS**
TITLE-ABS-KEY((football OR soccer) AND (youth OR young OR player OR athlete) AND ("motor performance""physical performance") AND longitudinal)
