# Supplementary material for: Motor Performance in Male Youth Soccer Players: A Systematic Review of Longitudinal Studies
Source: Sports (Basel). 2021 Apr 19;9(4):53. doi: 10.3390/sports9040053 (PMC8072970; doi:10.3390/sports9040053)
Supplement: Supplementary file 1 [file sports-09-00053-s001.zip › Electronic Supplementary Material Table S2. MaryamA.pdf]

Electronic Supplementary Material Table S2. Scores attributed to each study according to twelve criteria used in evaluating methodological quality

Citied	Study	Q1	Q2	Q3	Q4	Q5	Q6	Q7	Q8	Q9	Q10	Total (∑)	Percent	Quality
17	(Philippaerts et al., 2006)	2	2	0	1	1	1	2	2	0	2	13	65%	[moderate]
18	(Huijgen et al., 2010)	2	1	1	2	2	2	2	0	2	0	14	70%	[moderate]
48	(Mirkov et al., 2010)	2	1	1	2	2	1	2	2	2	2	17	85%	[high]
40	(Roescher et al., 2010)	2	1	0	2	2	2	2	2	0	2	15	75%	[high]
39	(Williams et al., 2011)	2	1	1	2	2	2	2	2	0	2	16	80%	[high]
45	(Gonaus & Muller, 2012)	1	1	0	2	2	2	2	2	2	2	16	80%	[high]
25	(Valente-dos-Santos, et al., 2012)	2	2	0	2	2	2	2	2	2	2	18	90%	[high]
19	(Valente-dos-Santos, et al., 2012)	2	1	0	2	2	2	2	0	0	0	11	55%	[moderate]
26	(Valente-dos-Santos, et al., 2012)	2	1	1	2	2	2	2	2	2	0	16	80%	[high]
27	(Valente-dos-Santos, et al., 2012)	2	2	1	1	2	2	2	2	2	2	18	90%	[high]
41	(Huijgen et al., 2013)	2	1	0	2	2	2	2	2	2	2	17	85%	[high]
42	(Carvalho et al., 2014)	2	1	1	2	2	2	2	2	2	0	16	80%	[high]
31	(Deprez et al., 2014)	2	2	0	1	2	2	2	2	2	2	17	85%	[high]
28	(Valente-dos-Santos, et al., 2014)	2	2	0	2	2	2	2	2	1	2	17	85%	[high]
29	(Valente-dos-Santos, et al., 2014)	2	2	0	2	2	2	2	0	1	2	15	75%	[high]
36	(Wrigley et al., 2014)	2	1	2	1	2	2	2	2	2	2	18	90%	[high]
24	(Bidaurrazaga-Letona et al., 2015)	2	1	0	2	1	2	2	2	0	2	14	70%	[moderate]
32	(Deprez, et al., 2015)	2	1	0	2	2	2	2	2	2	2	17	85%	[high]
33	(Deprez, et al., 2015)	2	2	0	1	2	2	2	2	0	2	15	75%	[high]
34	(Deprez, et al., 2015)	2	2	0	2	2	2	2	2	2	2	18	90%	[high]
46	(Forsman et al., 2016)	2	2	0	2	2	1	2	2	2	2	17	85%	[high]
20	(Francioni et al., 2016)	2	1	2	1	2	2	2	2	0	0	14	70%	[moderate]
21	(Zuber et al., 2016)	0	2	1	2	1	2	2	0	2	2	14	70%	[moderate]
43	(Carvalho et al., 2017)	2	1	1	1	2	2	2	1	2	2	16	80%	[high]
35	(Fransen et al., 2017)	2	1	0	1	2	1	2	2	2	2	15	75%	[high]
22	(Rebelo-Goncalves et al., 2017)	1	2	0	2	2	2	0	1	0	2	12	60%	[moderate]
44	(Francioni et al., 2018)	2	2	1	1	2	2	2	2	2	0	16	80%	[high]
39	(Coutinho et al., 2018)	2	2	1	2	2	2	2	2	2	2	19	95%	[high]
47	(Leyhr et al., 2018)	2	2	2	1	2	2	2	0	1	2	16	80%	[high]

23	(Bennett et al., 2019)	1	2	1	1	2	1	1	1	2	2	14	70%	[moderate]
37	(Moran et al., 2020)	1	2	2	2	1	2	2	2	2	2	18	90%	[high]
38	(Saward et al., 2020)	2	2	0	2	2	2	2	2	2	2	18	90%	[high]
